# Developing generic clinical trial animated explainer videos in the UK: results of a survey and case study

**DOI:** 10.1186/s13063-024-08687-5

**Published:** 2025-01-21

**Authors:** Clare Calvert, Vicki S. Barber, Duncan Appelbe, Kirsty Sprange, Claire Nollett, Fiona Lugg-Widger, Samantha Tanner, Duncan B. Richards

**Affiliations:** 1 Oxford Clinical Trials Research Unit, University of Oxford, Oxford, UK; 2 Nottingham Clinical Trials Unit, University of Nottingham, Nottingham, UK; 3 Centre for Trials Research, Cardiff University, Cardiff, UK

## Abstract

**Background:**

Animated short videos used to explain a concept or project are often called animated explainer videos (AEVs). AEVs can supplement or provide an alternative to participant information sheets as a means of giving information about clinical research to potential participants. Current use of AEVs tends to focus on the specifics of a particular trial, yet there are many common aspects of clinical research regardless of the interventions being investigated that can be poorly covered in current trial materials. The EXPLAIN initiative aimed to determine the top generic clinical trial topics considered most important by different UK trial stakeholders. The top three topics were then turned into AEVs and have been made freely available for use.

**Method:**

A list of generic clinical trial topics which often need explaining to potential trial participants when they are approached to take part in research was developed. Using a two-round Delphi survey of stakeholder groups (trial participants, patients, members of the public, site staff and clinical trials unit staff), the list of topics was expanded and prioritised to identify the topics most in need of clear explanation. The top three topics formed the basis of three AEVs, co-developed with patient and public partners.

**Results:**

Two hundred twenty-eight responses were received to the first round of the Delphi survey, and 167 of these respondents also completed the second round of the survey. The three topics prioritised for creation of animated explainer videos were as follows: (1) What is consent? (2) Who decides what treatment I get/What is randomisation? (3) Is it safe to take part in a trial/How do you know a trial is safe? Following virtual meetings with patient and public partners recruited from the Delphi respondents, a script for each AEV was co-produced before being developed into an AEV by a company specialising in animated video production.

**Conclusion:**

There are a wide range of generic concepts in which the use of animated explainer videos could be useful to improve participant understanding of clinical research. Via consensus survey across multiple stakeholders, we have determined a hierarchy of the importance of explaining these concepts. We envisage that the three AEVs created from this project will form the basis of a readily accessible library of animations to be utilised by trialists.

**Supplementary Information:**

The online version contains supplementary material available at 10.1186/s13063-024-08687-5.

## Background

Efficient trial delivery is key to being able to answer research questions. One of the key components of trial delivery is participant recruitment and retention. Information and terminology that can be delivered in a clear manner supports understanding, potential recruitment, and retention for those who consent to take part. Ensuring that there is high-quality clear information to support researchers to convey what might be unknown terms and concepts to potential participants helps to facilitate this. By giving individuals clear, unambiguous information on concepts they may have never encountered before this enables them to make informed decisions about whether to participate in a trial or not. It is an absolute requirement that individuals who decide to participate and consent to take part in health research understand and are fully informed about what is being asked of them, what will happen to them and their data, and any risks and benefits [1].

Digital technology is being increasingly used in all areas of health care. There is a wealth of published evidence supporting the effectiveness of audio-visual information about trials to improve patient understanding but not necessarily recruitment [2–5]; further research is needed regarding the wider usage of digital technologies in trials to provide information to participants [6, 7]. Clinical trials often involve terminology that is unfamiliar to most people, and there is frequent criticism that informed consent documents are overly long and technical in their language [8]. Researchers globally have started to develop and assess different messaging strategies that could help potential participants better navigate and understand information about clinical trials [9]. Animated explainer videos (AEVs) are one such approach. AEVs are short digitally created animated videos used to explain a concept pictorially, typically accompanied by voiceover, with the intent to communicate in a more engaging and accessible way [10, 11]. AEVs are increasingly used across clinical trials [12, 13] with evidence that they are an effective educational tool in a range of settings, including delivery of effective patient education in health contexts [14, 15].

The EXPLAIN initiative aimed to understand the usage of AEVs across the UK academic clinical trials unit network [16], to develop an agenda for a priority list of generic AEVs that the UK trials community would welcome and to then produce the top three AEVs.

## Method

To determine which were the main generic trial concepts that would benefit from the production of an AEV, we consulted the clinical trials community with the aim of arriving at a consensus. We used a modified Delphi technique commonly used in the health care community to gather consensus across multiple stakeholders [17–21]. An initial survey was created for round 1, and then for round 2, the survey was annotated with how an item had been rated previously by an individual and also across the different stakeholder groups. We did not hold a consensus meeting to discuss the final ratings given in round 2 of the survey. All surveys were accessible and displayed in the Research Electronic Data Capture (REDCap) tool [22, 23] hosted at the University of Oxford.

Ethics approval was sought from the University of Oxford Central University Research Ethics Committee (CUREC) to conduct this survey (Ref: R81465/RE001). The survey was entirely unrestricted in its access and open to anyone aged 16 years or older. Prior to starting the survey, a Participant Information Sheet (PIS) was displayed on screen and individuals clicked a box to confirm their age was 16 or older and to indicate their consent to take part (see Appendix 1 for the study participant information sheet).

The survey was targeted at four key groups that included staff, Patient and Public Involvement (PPI) partners, and anyone who had previously been approached to consider taking part in a trial. Table 1 further describes the four groups.

**Table 1:** Groups targeted to participate in the Delphi survey

Group approached	How members of the group were approached
Staff involved in the design, management, and conduct of clinical trials within the UK Clinical Research Collaboration (UKCRC) registered Clinical Trials Unit (CTU) network	Staff at UKCRC registered CTUs were emailed information about the project and a link to the PIS via the UKCRC Secretariat
PPI partners associated with UKCRC CTU Network CTUs	PPI partners were primarily reached via direct approaches by their associated CTUs. The CTUs sent a standardised email about the EXPLAIN initiative together with a link to the participant information to PPI partners
Staff at sites involved in the recruitment of clinical trial participants	All of the 53 UKCRC registered CTUs were sent an email on behalf of the EXPLAIN team asking them to pass onto staff at participating sites of trials run by their CTUs
Anyone that had taken part in a clinical trial or previously been approached about taking part in a clinical trial	UKCRC CTUs were asked to disseminate information about the project on their relevant social media accounts and to any trial participant contacts they had with permission to contact about other studies

Emails and social media posts containing a link to the survey were distributed in February 2023 (see Appendix 2 for the social media advert). The advertisements used on the social media platform X (previously Twitter) were available in English, Welsh, Polish, Panjabi, and Urdu. These languages were chosen using the 2011 census data [24] which identified Polish, Panjabi, and Urdu as the top three languages spoken in the UK other than English in England and Welsh in Wales. However, due to limited resources, the survey itself was only available in English. The team advertised the survey widely to maximise engagement across the clinical trials community. This included but was not limited to clinical trials units, national trial management groups, the NIHR Research Design Service (RDS), UK Trial Manager’s Network (UKTMN), and clinical research networks. A full list of those contacted and asked to distribute the survey link is included as Appendix 3.

### Modified Delphi survey—round 1

Round 1 of the Delphi survey (Appendix 4) was devised by a small working group from three academic clinical trials units (CTU) in the United Kingdom (UK), consisting of methodologists, trialists, and qualitative researchers. They discussed common trial concepts with colleagues and reviewed participant information sheets to identify an initial list of 20 concepts that might benefit from an AEV. These included ‘what is a clinical trial?’ and ‘why are trials blinded?’. These topics were entered into the online survey tool (REDCap) which was reviewed and edited by a patient with expertise in public involvement, to improve clarity and accessibility.

Individuals were asked to rate each of the 20 concepts for importance in terms of being made into an animated explainer video that could be used for most clinical trials, using an 11-point Likert scale (0–10) with 0 being ‘not important at all’ and 10 being ‘very important’. Participants were able to select ‘unsure’ if they were unable to give an opinion/did not understand the concept that they were being asked to rate. Participants could add additional comments on any of the concepts and were also invited to suggest additional generic trial concepts that could be developed into an AEV. Participants were asked in what capacity they were answering the survey to determine differences between groups in their responses. Any participants that worked in/were involved in trials were asked to select from a list of categories to describe their trials experience/exposure.

Round 1 of the Delphi survey opened on 10 February 2023 for 4 weeks. A response to the survey was recorded in REDCap as soon as the survey had been started by a participant, and therefore the total number of responses received included incomplete responses to the survey. The study team considered a complete response to the survey to be any response where the participant had rated all of the 20 topics presented in the survey.

The results were exported into Microsoft Excel™. The EXPLAIN team reviewed all the comments and extracted all the additional topics proposed which were grouped into themes to identify any new topics to be included in the second round of the Delphi survey. This resulted in an additional nine topics. A summary list of the additional topics suggested by participants along with the grouping/summation of the topics that was made by the EXPLAIN team is included as Appendix 5.

### Modified Delphi survey—round 2

For round 2 of the survey, individuals were asked to re-rate the 20 topics and provide a rating for the additional nine proposed topics. Participants were shown how they had individually scored each topic in round 1 and how others had scored each topic (mean topic score by participant category). Participants were then asked if they wished to change their original score for each topic. For the additional nine topics, participants were presented with what respondent group the suggestion had originated from. Screenshots from round 2 are included as Appendix 6.

Round 2 of the survey opened on 27 April 2023 for 4 weeks and was sent to the 228 participants who had provided a complete response to round 1. In error, it was also sent to the 63 that had entered a partial response to round 1; 20 of these respondents replied to round 2.

### PPI AEV development working groups

Individuals who responded to the Delphi survey as either a trial participant or as a member of the public were asked whether they wished to be involved in developing one of the animated explainer videos. Individuals that expressed an interest were advised of the top three topics identified by the Delphi survey and invited to join a PPI working group. Individuals were also asked to select their preferred meeting time and dates, with both daytime and evening times offered. When forming the PPI groups, individual preferences for both topic choice and meeting times were accommodated where possible, whilst also ensuring each PPI group had a mixture of professional PPI partners as well as individuals who had either participated in or been approached about taking part in a trial. Three PPI groups, each with between five and eight PPI partners, were formed and each group led by a member of the EXPLAIN study team experienced in public involvement.

An initial virtual meeting was held with each group to give an overview of the EXPLAIN project and the remit of the group. Each group was asked to decide what information was important to include in an AEV on their chosen topics and contribute to developing a script for the video.

The EXPLAIN study team identified some key terms associated with each topic and during the PPI meeting invited group members to share their views about these to encourage initial discussion. Members were also asked to share what they felt was important to include in the AEV and advise on best approach to presenting the topic information. Following the initial meeting, the PPI group leads prepared draft scripts for each AEV based on the feedback from their group and this was circulated to the members of their PPI group for review. Following further rounds of review, a script was agreed with each PPI group for the chosen AEV and sent to an external company experienced in AEV production to develop into an AEV by initially producing a storyboard, some static images, and a provisional voiceover script for review. Through emails, the PPI members were then asked to provide their thoughts on draft storyboards which were then used to ultimately create the AEVs. Note all PPI members were offered recompense for their time at the latest PPI approved rates [25].

## Results

The first round of the Delphi survey resulted in 291 responses recorded in REDCap of which 228 of these were complete responses and included in the analysis. The other 63 records were incomplete responses and were excluded from the final dataset. Participants were asked in what capacity they were answering the first round of the Delphi survey; participants were able to select more than one answer; broad summary categories were created by the study team from the responses given to this question (Appendix 7) to present the responses by a participant type in the second round of the Delphi survey. 

The mean rating of each topic was then calculated for each participant category.

Table 2 shows the responses to the survey by participant category.

**Table 2:** Responses to round 1 and round 2 of the Delphi survey by participant category

Participant category	No. responses (round 1)	No. responses (round 2)
Members of staff at a clinical trials unit	105	79
Members of site research teams	52	33
PPI partners	22	20
Clinical trial participants	20	14
PPI partners who have also taken part in a trial	19	14
Other	10	7
**Total**	**228**	**167**

For the 10 participants in round 1 that selected ‘other’, they stated their trials experience/exposure to be ‘R&I Manager’—Quality and Governance, a member of the Wales Cancer Network, a Patient and Public Involvement Lead, someone who works with Universities who complete trials or research, a PPI panel member for a trust and has previously worked in study recruitment and organisation, a member of a university department, a nurse interested in becoming a research nurse, a consumer member for the National Cancer Research Initiative (NCRI), a research champion, and an individual who has not participated in a trial but would have liked to.

For the members of staff at a CTU, responses came from a variety of individuals which are listed in Table 3. For those CTU staff that selected their CTU role as ‘other’, seven reported trial management team roles (data clerk/trial coordinator/data manager/ ‘CTSO’); other roles reported were clinical research nurse, administrative, and participant/REC member.

**Table 3:** CTU staff breakdown

Trial manager (or equivalent role)	41
Member of senior CTU/operational team	14
Other (see below)	11
Trial statistician	10
Clinical trial administrator	7
Data manager (or equivalent role)	6
Academic/methodologist	4
Quality assurance (QA) or safety team	3
Trial programmer	3
Not answered	3
Qualitative researcher	2
Chief investigator	1
**Total**	**105**

The mean importance scores reported by participant group on each topic during round 1 are listed in Table 4.

**Table 4:** Mean topic scores for round 1 of the survey by each participant category

	**Members of staff at a CTU**	**Members of site research teams**	**PPI partners**	**Clinical trial participants**	**PPI partners who have taken part in a trial**	**Other**	**All answers combined**	**All patient and public contributor answers combined**
Is it safe to take part in a trial/how do you know a trial is safe?	8.7	9.4	9	9.3	8.4	9.5	9	9
What is assent?/Why is assent needed?	8	8.4	8.8	8.7	8.8	9.4	8.3	8.9
What is consent?/Why do I have to give consent?	8.7	8.8	8.6	8.7	9.1	8.6	8.7	8.8
If I take part, what might happen to me?	8.5	9.2	9.1	8.6	8.2	9.5	8.8	8.6
What is a clinical trial?	8.8	8.9	9	8.5	8.6	8.2	8.7	8.6
Why do you need to do clinical trials?	8.8	8.5	8.4	8.5	8.6	8.5	8.6	8.5
Who decides what treatment I get? What is randomisation?	8.6	9.1	8.9	8.1	8.9	7.7	8.7	8.5
How will taking part in a trial impact my future healthcare?	8.2	8.7	8.7	8.3	8.1	9	8.4	8.5
What are my legal rights?	7.9	8.4	8.2	8.6	8.3	8.6	8.1	8.4
How do you know if a treatment works?	7.9	8.3	8.5	8.9	7.8	8.5	8.1	8.4
What is a placebo and why are placebos used?	8	9	8.4	8.6	8	7.9	8.3	8.3
What is a blinded trial?/Why are trials blinded?	7.8	8.4	7.7	7.8	8.5	8.7	8	8.1
How do you decide you can take part in a clinical trial?/How do you decide who is eligible to take part?	7.5	7.5	7.8	8.6	8.1	8	7.7	8.1
What is meant by ‘screening’?	6.6	6.7	7.9	8.5	7.9	7.2	7.1	8
What happens to my data?	7.7	8.8	7.5	8.1	7.6	8.8	8	7.9
How do you decide what is tested in a clinical trial?	7.1	7.4	7.4	7.9	7.6	8.6	7.4	7.8
Why don’t you give everyone the same treatment?	7.9	8.6	7.8	7.8	8.5	7	8	7.8
What is an ethics committee?	6.6	7.2	7.8	7.5	7.5	8.4	7	7.7
What is a crossover trial?	6.6	7.3	7	8.4	7.8	8.4	7	7.5
How do you decide how many people take part in a clinical trial?	6	6.1	6.8	7.4	7.4	6.4	6.3	7

Mean importance scores for each group—the higher the score, the stronger the preference for the topic—scores available were 0–10

Eighty-two of the participants suggested an additional 111 topics—which are included as Appendix 5. The EXPLAIN team assessed 51 of the topics as too specific to consider for inclusion in a library of generic AEVs and one topic had already been covered in the first-round survey. The remaining topics were grouped into themes from which the authors identified nine additional topics for inclusion in the second round.

1. Can I change my mind and withdraw from a trial, and if I do, what happens?
2. What happens if I don’t take part?
3. What will happen to me when the trial ends and how are the results used?
4. Why is it important to collect all this data from me?
5. How do trials benefit others?
6. What are the phases of clinical trials?
7. How do patients and the public help to design clinical trials?
8. Why is it important that trials include people from diverse backgrounds?
9. What is a consultee/legal representative?

There were 167 responses in round 2 from the full responders to round 1, and the number in each category are presented in Table 2 alongside round 1. To determine which topics should be developed into AEVs, we sorted the list in Table 5 by the ranking for ‘All Patient/Public Participants’ (these were all respondents excluding CTU staff and site staff). We did this to enable the views of patients and current/former trial participants to take priority, given they are the target audience for the animations.

**Table 5:** Ranks for the importance of each topic taken from the mean scores from round 2 of the survey

	**Clinical trial participants (*N* = 14)**	**PPI partners (*N* = 20)**	**PPI partners who have participated in a clinical trial (*N* = 14)**	**Members of staff at a CTU (*N* = 79)**	**Members of site research teams (*N* = 33)**	**Other (*N* = 7)**	**All answers combined (*N* = 167)**	**All patient and public contributor answers combined (*N* = 55)**
What is consent?/Why do I have to give consent?	2	1	1	2	3	4	1	1
Is it safe to take part in a trial/how do you know a trial is safe?	1	4	5	3	1	1	2	2
If I take part, what might happen to me?	4	2	2	5	2	1	3	3
What is assent?/Why is assent needed?	4	5	8	11	11	3	10	4
How will taking part in a trial impact my future healthcare?	8	5	11	7	13	5 =	8	5 =
What will happen to me when the trial ends and how are the results used?	2	9	5	10	15	5 =	9	5 =
Who decides what treatment I get?/What is randomisation?	12	7	2	6	5	13	5	7
What is a clinical trial?	4	3	9	1	4	23	4	8
What are my legal rights?	4	9	18	13	18	5	13	9
How do you know if a treatment works?	14	14	15	14	17	18	12	10
Can I change my mind and withdraw from a trial, and if I do, what happens?	12	11	16	8	6	8	7	11 =
Why is it important that trials include people from diverse backgrounds?	15	8	4	9	16	25	26	11 =
What is a placebo and why are placebos used?	9	12	10	12	8	20	11	13
Why do we need to do clinical trials?	9	13	13	4	7	20	6	14
How do you decide who can take part in a clinical trial?/How do you decide who is eligible to take part?	9	20	16	17	20	8	17	15
What is a blinded trial?/Why are trials blinded?	18	16	5	15	9	8	14	16
How do trials benefit others?	19	15	13	20	13	11	15	17
Why don’t you give everyone the same treatment?	19	16	11	16	12	24	16	18
What happens to my data?	17	21	21	18	10	11	18	19
What is meant by ‘screening’?	15	19	19	24	24	25	23	20
Why is it important to collect all this data from me?	21	16	24	19	19	13	19	21
What is an Ethics Committee?	23	22	23	25	26	16	24	22
What is a crossover trial?	25	24	22	26	23	16	25	23
How do you decide what is tested in a clinical trial?	22	24	26	21	22	19	22	24
What are the phases of clinical trials?	24	27	27	29	28	13	27	25
What happens if I don’t take part?	26	26	24	23	21	20	20	26
How do patients and the public help to design clinical trials?	29	23	19	22	24	25	21	27
What is a consultee/legal representative?	27	28	28	27	27	28	29	28
How do you decide how many people take part in a clinical trial?	28	29	29	28	29	29	28	29

Ranking of the importance of topics by each group—topics rated as 1 were ranked most important

The top eight topics identified by the patient/public participants (Table 6) were then evaluated to see which topics should be made into AEVs by the EXPLAIN initiative.

**Table 6:** Top eight topics identifiers by the survey public and patient participants

Topic	Rank
What is consent?/Why do I have to give consent?	1
Is it safe to take part in a trial/how do you know a trial is safe?	2
If I take part, what might happen to me?	3
What is assent?/Why is assent needed?	4
How will taking part in a trial impact my future healthcare?	5 =
What will happen to me when the trial ends and how are the results used?	5 =
Who decides what treatment I get?/What is randomisation?	7
What is a clinical trial?	8

There was a consensus that although three of the topics would be very useful for potential participants to be able to understand a concept or impact of a trial, the animation would need to have specifics related to an individual trial to make it have any value for viewers. These would vary widely from trial to trial and would therefore not make a suitable subject matter for a generic explainer. Therefore, the topics ‘If I take part, what might happen to me?’, ‘How will taking part in a trial impact my future healthcare?’, and ‘What will happen to me when the trial ends and how are the results used?’ were not progressed. For the topic ‘What is assent?/Why is assent needed?’, although a generic AEV could easily be produced, the AEV would only apply to trials including minors, and as the aim of the EXPLAIN initiative was to produce generic explainers that would be able to be used by as many people as possible, producing an animation that would only be used by some trials was felt not to meet the ethos of using the small amount of funds secured to produce 3 animations. It was therefore decided to produce AEVs on ‘What is consent?’, ‘Is it safe to take part in a trial/how do you know a trial is safe?’, and ‘Who decides what treatment I get/what is randomisation?’. The subject of ‘is it safe to take part in a trial’ covers a wide range of subject matter, ranging from regulatory and ethical review through to specifics about the trial intervention, clinical trial oversight committees, and ongoing monitoring. As such, it was decided that a generic AEV on this topic would have to concentrate on the procedures that apply to all clinical trials regardless of the intervention, i.e. how a trial is reviewed and what checks are made throughout a trial rather than any specific interventions for a trial which might include blood tests, hospital stays, and clinic visits.

Forty-seven participants had expressed an interest and were invited to work together with the EXPLAIN team to shape the information given to clinical trial participants in the three AEVs subsequently developed. Of these, three groups with a total of 19 participants were formed.

## Discussion

There is clear overlap in all trials of the core concepts that underlie them, such as in their set-up, conduct, and reporting. Four of the topics out of the 29 asked about in round 2 were ranked as number one importance by one or more of the groups that took part in the study—‘what is consent?/why do I have to give consent?’, ‘is it safe to take part in a trial/how do you know a trial is safe?’, ‘if I take part, what might happen to me?’, and ‘what is a clinical trial’. As these are the questions that anyone would ask if approached about participating in research, they were not surprising. There were however three topics that stood out as being rated in the top five by one of the study groups but not in the top five of any of the other groups. These were ‘why do we need clinical trials?’, rated in the top five by the members of CTU staff; ‘why is it important that trials include people from diverse backgrounds’; and ‘what is a blinded trial?/why are trials blinded?’, rated highly by the PPI partners that had participated in a trial. Staff within a CTU regularly review screening logs, which often record the reasons for exclusion and as a result may have flagged the need for clinical trials because they observe a lack of understanding of why trials are needed and interventions/treatments are not just brought into clinical practice. The two topics raised by the PPI partners that had participated in a trial are generic topics; however, they did not align with the ratings of trial participants that had not been a PPI partner. The greater exposure to a trial team, and topics raised, and national initiatives might explain why these topics were rated by this group.

Within the trials community, we should be working smarter, to not be consistently repeating time producing materials that have been already produced. With the ever-tightening financial climate and rising costs, being able to focus resources on developing reusable and freely available tools for use within the trials community will allow funds to then be better used for more trial specific activities. This research has collated what those concepts are in relation to potential participants and how we can best support participation and retention in trials through provision of clear and concise information through AEVs. The EXPLAIN team have co-produced three AEVs with PPI individuals who participated in the survey. The animations are accessible from the EXPLAIN website https://explain.octru.ox.ac.uk.

## Limitations

The study was widely advertised to the clinical trials community through several routes. Whist the survey received a good response rate, the views of only a small proportion of all staff and patients associated with trials research are represented. As a study team, we tried to ensure the advertisement of the study in multiple languages; however, following completion of the survey, it was noted that in the 2021 census, Romanian had replaced Panjabi as the second most common language spoken in the UK other than English in England and Welsh in Wales, and we had not put out an advert for our study in Romanian. It may be that other topics would have been prioritised with a wider sample. However, the survey provided a good starting point for prioritising and creating the first three AEVs in what we envisage will be a library of free resources.

## Conclusion

A modified two-round Delphi study has created a prioritisation list of trial stakeholder opinions for those trial-related topics that would be most suitable in being produced as generic animated explainer videos to aid trial understanding in the UK. The three AEVs produced as part of the EXPLAIN project are available at https://explain.octru.ox.ac.uk.

## Supplementary Information

Supplementary Material 1: Appendix 1: Study participant information sheet.

Supplementary Material 2: Appendix 2: The social media advert.

Supplementary Material 3: Appendix 3: Full list of those contacted and asked to distribute the survey link.

Supplementary Material 4: Appendix 4: The survey used.

Supplementary Material 5: Appendix 5: Summary list of the additional topics suggested by participants along with the grouping/summation of the topics that was made by the EXPLAIN team.

Supplementary Material 6: Appendix 6: Screenshots from round 2.

Supplementary Material 7: Appendix 7.

## Data Availability

The survey used is available as Appendix 4 to the paper. The datasets used and/or analysed during the current study are available from the corresponding author on reasonable request.

## Abbreviations

AEV: Animated explainer videos
CTU: Clinical trials unit
EAs: Explainer animations
NCRI: National Cancer Research Initiative
PPI: Patient and Public Involvement
PIS: Participant Information Sheet
REDCap: Research Electronic Data Capture
UK: United Kingdom
UKCRC: United Kingdom Clinical Research Collaboration
US: United States
