# Supplementary material for: Developing generic clinical trial animated explainer videos in the UK: results of a survey and case study
Source: Trials. 2025 Jan 21;26:25. doi: 10.1186/s13063-024-08687-5 (PMC11753093; doi:10.1186/s13063-024-08687-5)
Supplement: Supplementary file 1 — Supplementary Material 1: Appendix 1: Study participant information sheet. [file 13063_2024_8687_MOESM1_ESM.docx]

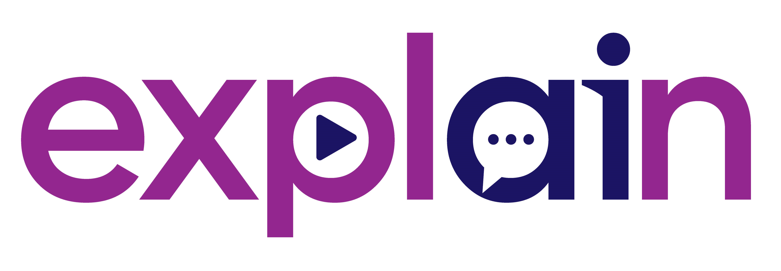


Appendix 1 – Delphi survey Information Sheet

**The EXPLAIN initiative**

**CUREC Approval Reference: R81465/RE001**

# Introduction

The aim of the EXPLAIN initiative is to develop short videos to explain key aspects of clinical trials to help patients make an informed decision about whether to take part in a clinical trial. The explainer videos produced by this initiative will be made freely available to Clinical Trials Units (CTUs) that are part of the UK Clinical Research Collaborative (UKCRC).

We are seeking the views of those who take part in clinical trials (trial participants) and those involved in designing, managing, and recruiting to clinical trials to help identify the key topic areas for which explainer videos should be created. This information will then be used to start the creation of a library of explainer videos that will be freely shared with UKCRC registered CTUs to utilise in and across their portfolio of trials as each trial/ CTU decides to.

# Why is this research being conducted?

It is important that everyone who might be suitable for a clinical trial is given the opportunity to learn and understand what it is about, to be able to then consider if they wish to participate.

Traditionally, information about trials is provided as paper leaflets or as a webpage on the internet. Leaflets are not always the best way of making sure that things are understood, many people prefer visual information (animations or short videos). One way of providing information is by using animations that clearly explain things – these are called explainer videos.

#
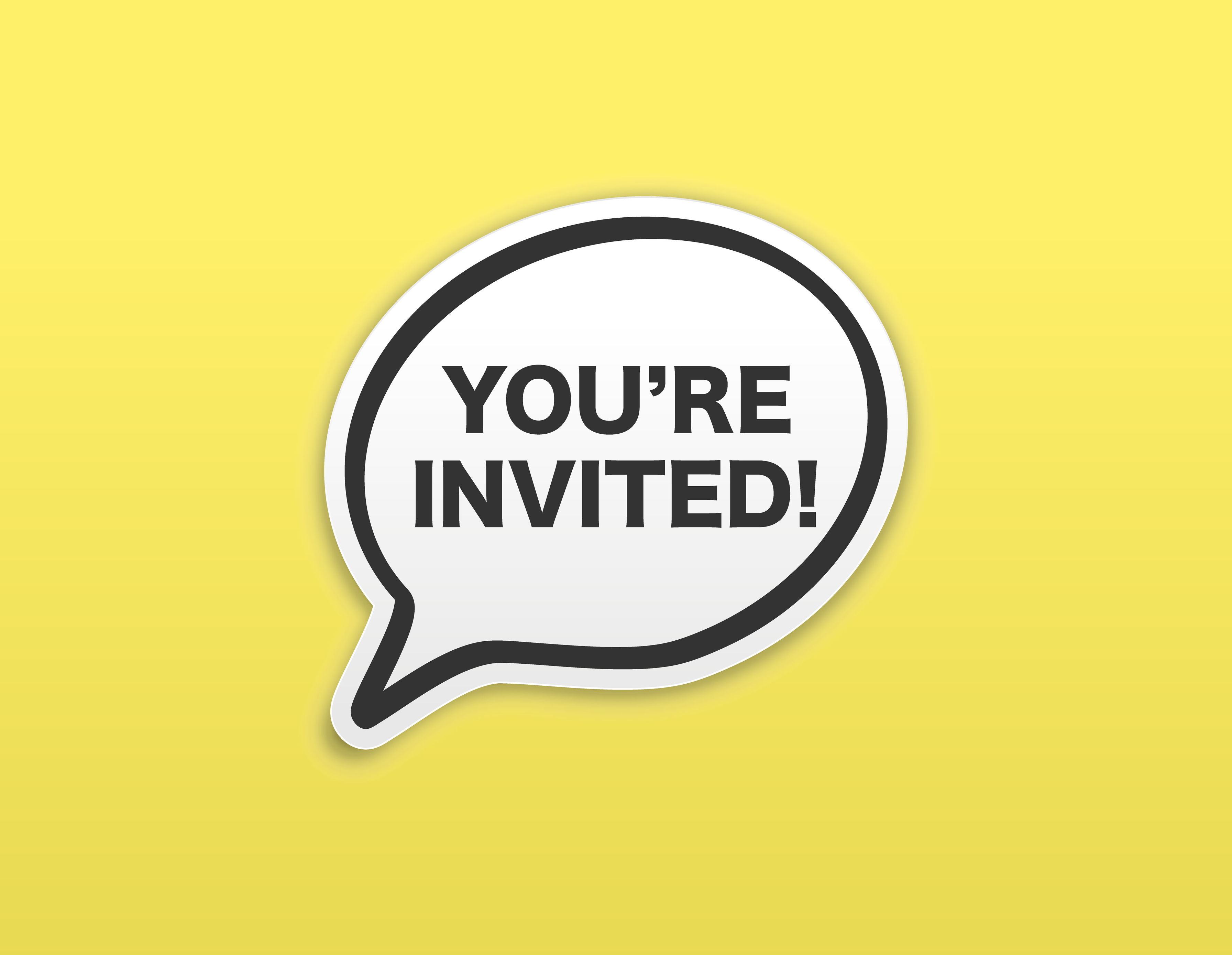
Why have I been invited to take part?

You have been invited to take part as you are either a participant who has taken part in a clinical trial in the past or currently, you work in a clinical trials unit or have a role in seeking informed consent from potential clinical trial participants. We also would like people approached about trials but who decided not to take part to be part of the EXPLAIN project. We would like to hear your views about what explainer animations should be produced by the EXPLAIN initiative. To take part, you must be aged 16 years of over.

# What will I need to do if I take part in this research?

If you agree to take part in the EXPLAIN study, we will ask you to complete some online questionnaires as part of what is known as a Delphi Survey. A Delphi survey is a multi-stage process which is designed to reach a group decision or ‘consensus’ on a topic. Each questionnaire will be designed based on the answers given by participants to the previous questionnaire.

We expect the EXPLAIN Delphi survey to consist of 2 rounds:

**Delphi round 1:** In this round we will list a number of clinical trial topic areas for which an animated explainer video might be useful and ask you to rate the importance of each of these from not important at all to very important. You will also have the option to explain your ratings, as well as suggest any additional topic areas to be considered.

**Delphi round 2:** In this round, you will be provided with a summary of the findings of the round 1 survey and asked to rank the topics identified by round 1 by order of importance. We will also ask some additional questions about any prior use of explainer videos.

It is expected that each round of the Delphi survey will take no more than 10 minutes to complete.

We will collect your e-mail address during round 1 of the Delphi survey so that we can later invite you to complete a further questionnaire in round 2 of the survey.

We will contact you by e-mail regarding round 2 as soon as this is available to complete. It is not anticipated that we will require a 3^rd^ round, however we will contact you by e-mail to let you know if a third round is necessary and the anticipated dates of this.

# Do I have to take part?

No. It is up to you to decide whether to take part. You can decide to stop replying to the questionnaires or by contacting us at [explain@ndorms.ox.ac.uk](mailto:explain@ndorms.ox.ac.uk) to ask to be removed from any email list.

You may ask any questions before deciding to take part by contacting the EXPLAIN study team (see contact details at the end of this information sheet).

If you withdraw from the study, we will not send you any further survey invites. If you withdraw before the Delphi survey is complete, we will delete your contact details, but we will keep anonymous research data that has already been collected.

# Who is conducting this research?

The lead for the research is Professor Duncan Richards, who is part of the Oxford Clinical Trials Research Unit (OCTRU) attached to the Nuffield Department of Orthopaedics, Rheumatology & Musculoskeletal Sciences (NDORMS) at the University of Oxford. This project is being completed in collaboration with researchers at the Centre for Trials Research, University of Cardiff and the Nottingham Clinical Trials Unit, University of Nottingham.

# How will my data be used?


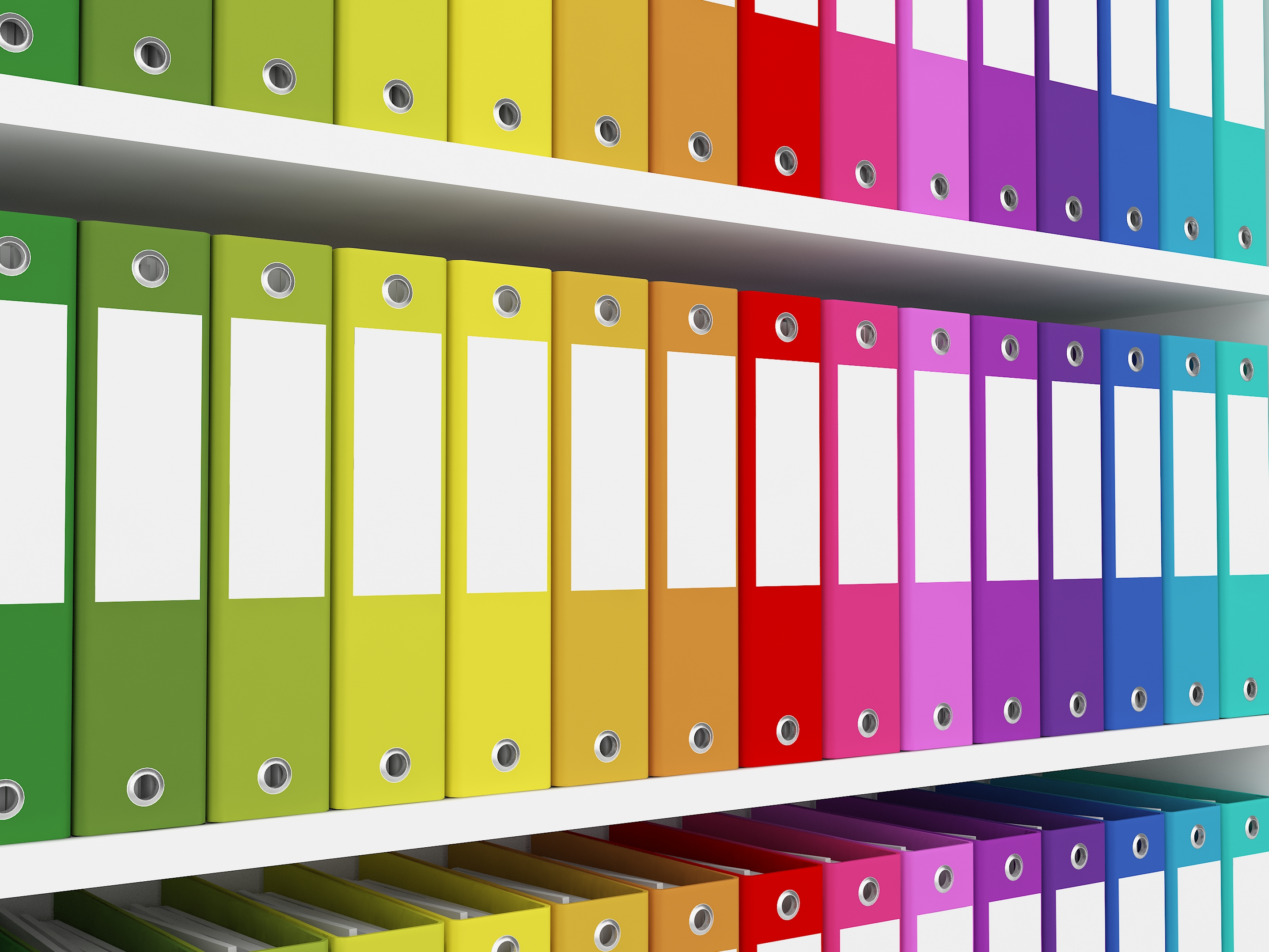
The responses you provide in each round of the Delphi survey will be stored in a password-protected electronic database on University of Oxford secure servers, which will be accessible only by the members of the research team at the University of Oxford.

In order to contact you regarding subsequent rounds of the Delphi survey we will need to collect your e-mail address. If you work at a CTU or have another role in identifying potential participants we will also collect information about your role. Email addresses will be stored until the Delphi survey has been completed. Other research data collected will be archived by the University of Oxford for a 3 years.

We will take all reasonable measures to ensure that data remain confidential.

The findings from the research will be written up and published in academic publications and may be presented at conferences. You will not be identified in any publication or report arising from this research.

# Who will have access to my data?

The University of Oxford is the data controller with respect to your personal data and, as such, will determine how your personal data is used in the study. The University will process your personal data for the purpose of the research outlined above. Research is a task that we perform in the public interest. Further information about your rights with respect to your personal data is available from <https://compliance.admin.ox.ac.uk/individual-rights>.

Anonymised results of the surveys will be shared with other collaborators on the project – members of the research team at the University of Cardiff and the University of Nottingham.

# Who is funding the research?

The EXPLAIN initiative is funded by the National Institute for Health Research (Supporting Efficient/innovative delivery of NIHR research 2022 (NIHR152363)).

# Who has reviewed this study?

This project has been reviewed by, and received ethics clearance through, a subcommittee of the University of Oxford Central University Research Ethics Committee R81465/RE001.

# Who do I contact if I have a concern, or I wish to complain?

If you have a concern about any aspect of this study, please contact the EXPLAIN study team:

E-mail: [EXPLAIN@ndorms.ox.ac.uk](mailto:EXPLAIN@ndorms.ox.ac.uk)

Tel: 01865 223469

We will acknowledge your concern within 10 working days and give you an indication of how it will be dealt with. If you remain unhappy or wish to make a formal complaint, please contact the Chair of the Medical Sciences Interdivisional Research Ethics Committee at the University of Oxford who will seek to resolve the matter as soon as possible:

Email: ethics@medsci.ox.ac.uk; Address: Research Services, University of Oxford, Boundary Brook House, Churchill Drive, Headington, Oxford OX3 7GB

**Thank you for considering taking part in the EXPLAIN initiative.**

**If you wish to take part, please click the link below to access the survey:**

[**https://explain.octru.ox.ac.uk/delphi.html**](https://explain.octru.ox.ac.uk/delphi.html)

**
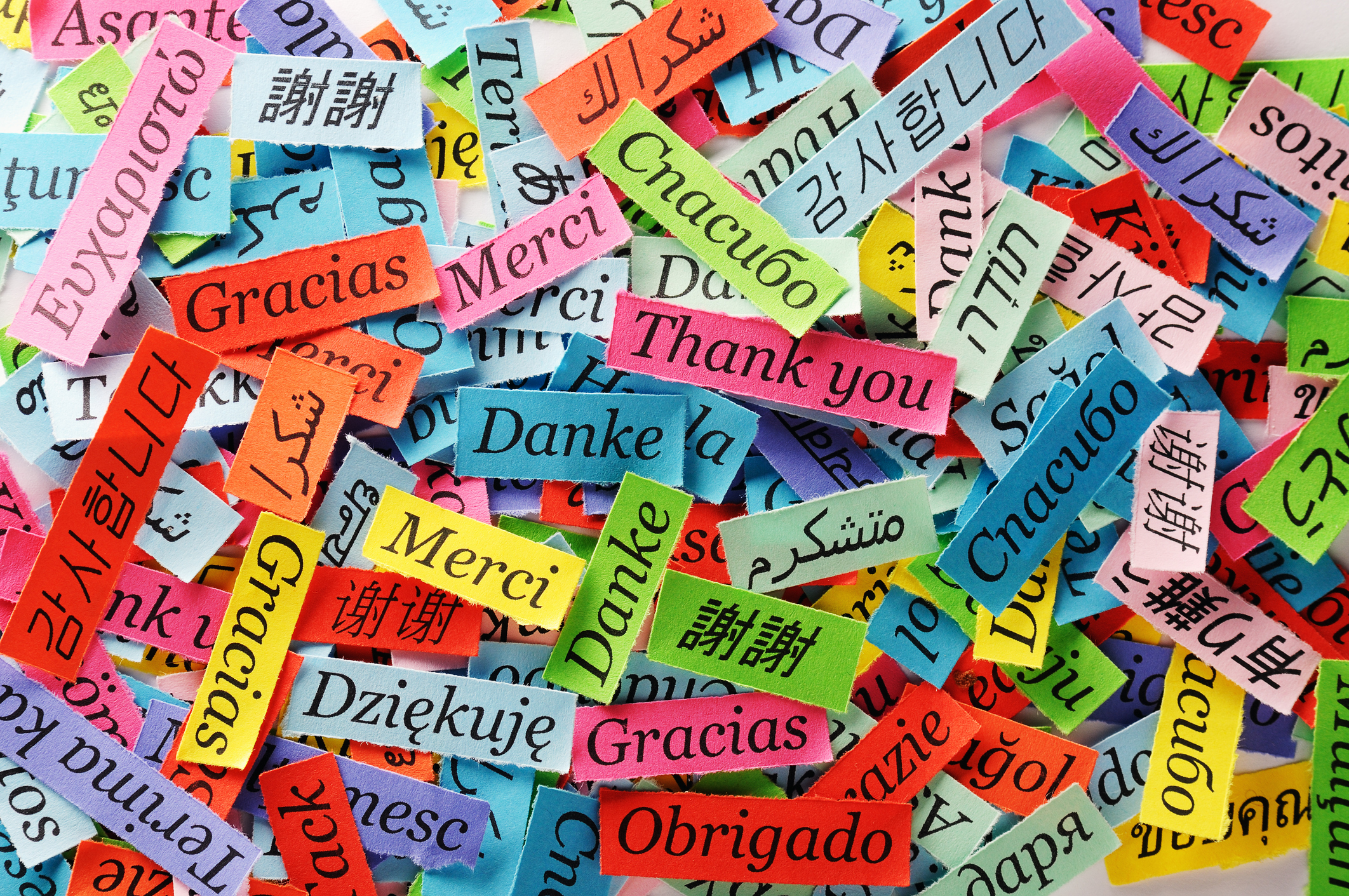
**

You can access up to date information on the EXPLAIN initiative by visiting our website: <https://explain.octru.ox.ac.uk/>
