# Supplementary material for: Developing generic clinical trial animated explainer videos in the UK: results of a survey and case study
Source: Trials. 2025 Jan 21;26:25. doi: 10.1186/s13063-024-08687-5 (PMC11753093; doi:10.1186/s13063-024-08687-5)
Supplement: Supplementary file 2 — Supplementary Material 2: Appendix 2: The social media advert. [file 13063_2024_8687_MOESM2_ESM.docx]

**Appendix 2 EXPLAIN: Advert for Delphi Survey**

**The EXPLAIN study team would like you to help shape the information given to clinical trial participants (The Explain Initiative -** [https://explain.octru.ox.ac.uk](https://explain.octru.ox.ac.uk/)**). See below for details.**

**To complete the survey:** <https://explain.octru.ox.ac.uk/delphi.html>


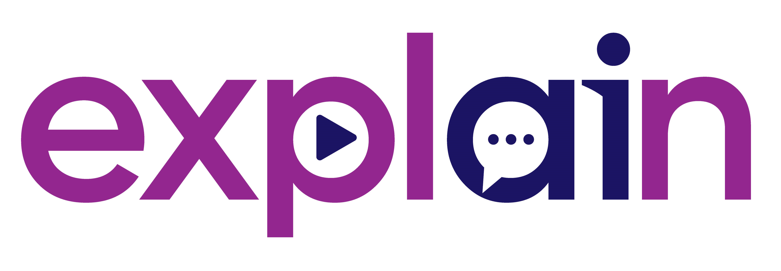


**The EXPLAIN study is being conducted by researchers at the University of Oxford, University of Nottingham, and Cardiff University.**

**We are looking to create some short videos/animations (that last no more than a few minutes) to help people understand more about terms used in clinical trial documents.**

**We would really like to hear from you if:**

- - - **You have taken part in a clinical trial (or been approached about one), or**
    - **You are Patient and Public Involvement Partner, or**
    - **You are a member of a site research team with a role in recruiting participants, or**
    - **You are a member of staff at a UKCRC registered Clinical Trials Unit (CTU)**

**We would love to hear your views about your experience and help us to shape the information given to people taking part in research studies in the future.**

**For further information and to take part in our short survey (10 mins) please visit:** <https://explain.octru.ox.ac.uk/delphi.html>

You can access up to date information on the EXPLAIN study by visiting our website: <https://explain.octru.ox.ac.uk/>

**Thank you for considering taking part in the EXPLAIN initiative**
