# Supplementary material for: Developing generic clinical trial animated explainer videos in the UK: results of a survey and case study
Source: Trials. 2025 Jan 21;26:25. doi: 10.1186/s13063-024-08687-5 (PMC11753093; doi:10.1186/s13063-024-08687-5)
Supplement: Supplementary file 3 — Supplementary Material 3: Appendix 3: Full list of those contacted and asked to distribute the survey link. [file 13063_2024_8687_MOESM3_ESM.docx]

Appendix 3 – List of groups contacted and asked to distribute/signpost the link to the EXPLAIN survey (Confirmation was not requested as to whether the link was distributed)

- UKCRC CTU Network – CTU Directors and Operations Directors to then forward to their teams and PPI partners
- UK Trial Managers Network (UKTMN)
- UK Research Design Service (RDS)
- Clinical Research Network (CRN)
- X(Twitter) posts were made by the Oxford Clinical Trials Research Unit from their account that was retweeted by 3 organisations - Nottingham Clinical Trials Unit, the UKCRC CTU Network and the Centre for Statistics in Medicine Oxford.
- All staff members within the Oxford Clinical Trials Research Unit (OCTRU)
- All staff members within the Nottingham Clinical Trials Unit (NCTU)
- All staff members within the Cardiff Clinical Trials Unit
- Newsletters from the Oxford University Hospitals NHS Foundation Trust, the Medical Sciences Division within the University of Oxford
- The Patient and Public Involvement Officer of the Nuffield Department of Orthopaedics, Rheumatology and Musculoskeletal Sciences asking to distribute to any PPI partners directly
