# Supplementary material for: Developing generic clinical trial animated explainer videos in the UK: results of a survey and case study
Source: Trials. 2025 Jan 21;26:25. doi: 10.1186/s13063-024-08687-5 (PMC11753093; doi:10.1186/s13063-024-08687-5)
Supplement: Supplementary file 4 — Supplementary Material 4: Appendix 4: The survey used. [file 13063_2024_8687_MOESM4_ESM.docx]

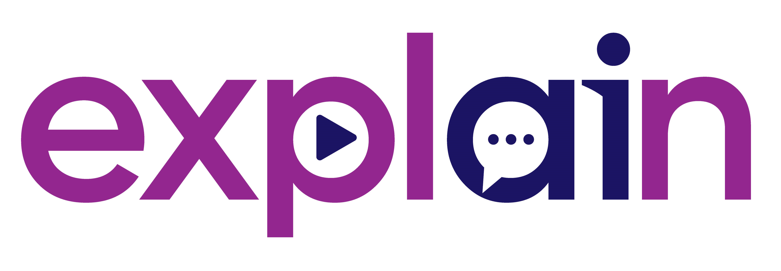


**Delphi Survey – Round 1**

**If you confirm that you have read the EXPLAIN participant information sheet and agree to participate with the understanding that the data (including any personal data) you submit will be processed accordingly, please tick the box below to start.**

☐ Yes, I agree to take part and confirm that I am aged 16 years or over

1. Are you answering this survey as: (participants may select more than one if applicable)
2. An individual who has participated in a clinical trial
3. An individual who was approached to take part in a clinical trial but decided not to take part
4. A PPI partner
5. A member of staff at a Clinical Trials Unit
6. A member of a site research team e.g. Principal Investigator/Research Nurse
7. Other. Please specify ________

If (1d) selected, display:

This survey is designed for completion by **individual CTU staff members** to obtain individual views on which generic explainer videos you would like to be produced by the EXPLAIN initiative.

Please note that a separate survey has been sent to CTU Directors to complete which will be used to collect information about the existence of explainer animations already used within your CTU.

Please answer this survey using your own opinions/views.

1. Please provide a contact e-mail address: __________________________________________

Please note this will be used to contact you for the second round of the Delphi Survey only. The answers you give will be kept confidential and you will not be identified in any results.

For the purposes of clarity and this survey - an explainer video is a video which helps people to understand more about taking part in a clinical trial. This may be a video or short animation (they are typically no more than a few minutes) that is given to you by the researchers conducting the clinical trial, or one that you find yourself e.g. through an online search.

<example explainer videos to be embedded into survey or as a link to explainer videos at <https://fame-info.digitrial.com/>>

1. Please consider these questions from the point of view of a clinical trial participant, please rate how important it is to you for the following clinical trial topics to be considered for development of animated explainer videos that could be used for **any trial.**

Please add any additional comments you wish to make about each topic choice/selection in the free text comments box.

0 = not important at all 10= Very important

|  | 0 | 1 | 2 | 3 | 4 | 5 | 6 | 7 | 8 | 9 | 10 | Unsure | Comments |
| --- | --- | --- | --- | --- | --- | --- | --- | --- | --- | --- | --- | --- | --- |
| Topic* |  |  |  |  |  |  |  |  |  |  |  |  |  |

*final topic list:

- What is a clinical trial?
- Why do we need to do clinical trials?
- How do you decide what is tested in a clinical trial?
- What is a blinded trial?/Why are trials blinded?

*A blinded trial in a trial where the participants and/or researchers do not know which drug or treatment is being given to which participant*

- What is a crossover trial?

*A crossover trial is a type of trial where participants receive two or more treatments but the order in which they receive them will be different amongst the participants.*

- How do you decide how many people take part in a clinical trial?
- Why don’t you give everyone the same treatment?
- Who decides what treatment I get?/ What is randomisation?

*Randomisation is a computer-generated method used to select the treatment a participant receives in a clinical trial*

- What is a placebo and why are placebos used?

*A placebo is a drug or treatment that has no active ingredient but is made to look the same as an active drug or treatment*

- What is consent?/Why do I have to give consent?

*Consent is a process in which potential participants are given important information about a clinical trial so that they can make an informed decision about taking part.*

- What is assent?/ Why is assent needed?

*Assent is used in clinical trials involving children/young people and is a process in which children are given easy to understand information about a clinical trial to help them decide if they want to take part.*

- How do you decide who can take part in a clinical trial?/How do you decide who is eligible to take part?

*Each clinical trial has a set of specific requirements (called eligibility criteria) that participants must meet to be able to take part. If a participant meets these requirements, they are considered ‘eligible’ to take part.*

- What is meant by ‘screening’?

*Screening in the context of research is a process used to determine if a patient is suitable to take part in a trial.*

- What are my legal rights?
- How will taking part in a trial impact my future healthcare?
- How do you know if a treatment works?
- If I take part, what might happen to me?
- Is it safe to take part in a trial/how do you know a trial is safe?
- What is an Ethics Committee?

*An Ethics Committee is a group of people who review clinical trials and ensure that the trial is ethical. They ensure that the rights, safety, dignity and wellbeing of research participants are protected.*

- What happens to my data?

1. Are there any additional topics not listed above that you would like us to consider when developing animated explainer videos? These should be topics that will help participants with their understanding of clinical trials better and what taking part in a trial involves.
2. Yes
3. No
4. Don’t know

If a) Please provide details:

For each topic that the participant has listed above they will be asked:

Please rate how important it is to you for the topic to be considered for development of animated explainer videos that could be used for any trial.

Please add any additional comments you wish to make about each topic choice/selection in the free text comments box.

1 = not important at all 10= Very important

|  | 1 | 2 | 3 | 4 | 5 | 6 | 7 | 8 | 9 | 10 | No opinion | Comments |
| --- | --- | --- | --- | --- | --- | --- | --- | --- | --- | --- | --- | --- |
| Topic** |  |  |  |  |  |  |  |  |  |  |  |  |

** any topics identified by participant in question 4.

1. If **Q1 = a, b or c,** the following questions will be asked:

If **Q1 = c,** the following additional question will be asked first:

How many years have you been involved in clinical trials as a PPI partner:

Less than 1 year

1-3 years

4-6 years

7-10 years

More than 10 years

How many trials (approximately) have you been approached to take part/consent to as a participant?

1-2

3-5

6+

How many trials (approximately) have you consented to take part in?

0

1-2

3-5

6+

If **Q1. = d**, the following questions will be asked:

Please select your role:

Trial Manager (or equivalent role)

Trial Statistician

Trial Programmer

Data Manager (or equivalent role)

Clinical Trial Administrator

Chief Investigator

Member of Senior CTU/Operational team

Quality Assurance (QA) or Safety team

Qualitative Researcher

Academic/Methodologist

Other, specify: __________________________________

How many years have been working in trials? (select one only)

Less than 1 year

1-3 years

4-6 years

7-10 years

More than 10 years

If **Q1. = e**, the following questions will be asked:

Please select your role:

Research Nurse/Midwife

Principal Investigator

Associate Principal Investigator

Trial coordinator

Other, specify: __________________________________

How many years have you been working in/recruiting to trials? (select one only)

Less than 1 year

1-3 years

4-6 years

7-10 years

More than 10 years
