# Supplementary material for: Developing generic clinical trial animated explainer videos in the UK: results of a survey and case study
Source: Trials. 2025 Jan 21;26:25. doi: 10.1186/s13063-024-08687-5 (PMC11753093; doi:10.1186/s13063-024-08687-5)
Supplement: Supplementary file 5 — Supplementary Material 5: Appendix 5: Summary list of the additional topics suggested by participants along with the grouping/summation of the topics that was made by the EXPLAIN team. [file 13063_2024_8687_MOESM5_ESM.docx]

**Appenidx 5 - ADDITIONAL TOPICS SUGGESTED IN EXPLAIN Delphi round 1 survey**

| Participants suggesting additional topics | n=82 |
| --- | --- |
| Additional topics suggested | n=111 |

| **Topic theme** | **Additional topics as suggested by participants** | **New topic(s) identified for second round Delphi** |
| --- | --- | --- |
| **WITHDRAWAL OR SAYING NO TO TAKING PART** | - What happens if I don't want to be in the trial any more? - withdrawl of consent at any time - Can I withdraw from a trial and if so, how? - Can I withdraw at any time? - What happens if I want to stop my participation in the trial? - Withdrawal from clinical trials - Withdrawal / change of consent - What happens to my care if I don't want to take part or take part and drop out - What happens if I say 'no' to taking part - What happens if I no longer wish or am able to take part in a study? - Legal rights and what happens to my data are covered but withdrawal needs to be covered as a topic on its own and what happens to care / data after this. | - Can I change my mind and withdraw from a trial, and if I do, what happens? - What happens if I don’t take part? |
| **END OF TRIAL/ TRIAL RESULTS** | - What happens after a trial ends? - What happens after the trial? - end of trial - What happens at the end of trials - What happens when study ends - results of trial - results of trial - How will the results of the study be used? - results of trial - How are the results of the trial used? - Results - Result - What happens after the trial is finished and i am being treated with this "new drug" can i still receive this after the trial is ended?* - what provision is there for after trial health care and or assessments* - Results of trial, will they be availlable, when, how* - How will I find out about the results of the trial?* - What are the potential impacts of a clinical trial. How can it change practices. | - What will happen to me when the trial ends and how are the results used? |
| **DATA COLLECTION/ QUESTIONNAIRES/ FOLLOW-UP** | - The Importance of Follow-up - completing diaries/questionnaires - Why are Participant questionnaires a useful tool for clinical research? - data collection process - Requirements for ling-term follow up, specifically for safety and why participant retention for this is so important - I'd suggest adding something about the importance of compliance with treatment and follow up - Importance of participants data within a clinical trial - Retention - Why is completing your [participant data collection tool type] important? - why are PROMs important - Data linkage - retention | - Why is it important to collect all this data from me? |
| **RISKS/BENEFITS** | - As part of questions what will happen to me and how do you know they are safe, "risks" of taking part should be mentioned - Will I benefit from participating? - What do I get from taking part if applicable - Risks and benefits of taking part - What are the benefits to being part of a clinical trial - Benefits of participation (to society, to people like yourself, to people less fortunate, etc) | - How do trials benefit others? |
| **TYPES OF TRIALS** | - Single/blind/opmne-label (in addition to double-blind) - Adaptive clinical trials - the difference between phases of study (ie, pre marketing vs post marketing authorisation) - What different type of trials are there? - Phase of development/stage along the pipeline - Timeline of study | - What are the phases of clinical trials? |
| **PUBLIC ROLE/PPI/EDI** | - Why mixed ethnic origins can be valuable to trials. - Value of patient/participant contribution - Who or what is PPI - 1) What is public involvement and engagement and where can I find further PPIE opportunities 2) Common differences between commercial versus academic research 3) Common restrictions and data collection requirements around contraception, pregnancy, and breastfeeding in drugs trials. 4) Why some studies collect samples and how these samples and associated data is used and regulated, e.g. HTA legislation. - Acknowledge historical abuses in clinical research as there is still mistrust especially amongst ethnic minority groups - Role of the public voice, especially PPI and plain English statements. | - How do patients and the public help to design clinical trials? - Why is it important that trials include people from diverse backgrounds? |
| **EXCLUDED TOPICS FOR NOT BEING GENERIC ENOUGH** | **Finance**   - participant payment/expenses - Can I get paid for being in a clinical trial? - Funding - Financial considerations - Who can fund clinical research (NIHR, Charities, Commercial funder)?   **TAKING PART**   - Why was I asked to participate? - Side effects - Who will be in charge of my care if I am on a clinical trial? - what is expected of me (participant) when taking part in this clinical trial? - the whole process of being involved in a clinical trial in a capsule - who to discuss details with - and HOW to approach them - No pressure to take part - Why i have been asked to take part   **Communication**   - Communication methods - How will I be kept up to date with how the trial is going, and the trial results - Let patient know that they can contact the trial staff at any point to discuss participation options if any personal issue arise - Support for individuals taking part and how it is done..one person to contact, reward of time. Help with WiFi or whatever for meetings etc   **Samples**   - Donating samples for clinical trials - What will happen to samples I donate during the research and afterwards - Use of biological samples donated by patients - Donating samples OR data to future research   **Miscellaneous**   - The Future - Ease of use - captioned/subtitled videos - Emergency medicine - Clinical trials in emergency circumstances - adherance - translated videos into other languages - Pressure / Coercion - Clarification - Consistency of language - How can i take part in the design of trials? - About the intervention - Usual length of a study - Clinical trials in paeds - All question - Travel - language interpretation and inclusitivity - visuals in animation - Previous trial results - Whether the trial has any limitations, eg to those who can use specific technology or have English as their first language - Family and Friends - adverse events and changes in medication - being aware that English may not be the participants first language - Suitability for paediatric patients and for patients who are visually impaired or have hearing impairments. Studies should be available to all. - Explain the length of time the study takes and what participant will need to do - If the trial is always face to face or email or telephone call - Time commitment - what is the placebo effect? what happens if my symptoms have improved and I've been taking a placebo? - Equipoise - International trials - including UK participants | N/A |
| **MISCELLANEOUS** | - Consultees / legal representatives | - What is a consultee/legal representative? |
| **COVERED BY EXISITING TOPICS** | - What is a clinical trial? | N/A |

*Was not considered a generic topic but falls under main topic theme
