# Supplementary figures and images for: Developing generic clinical trial animated explainer videos in the UK: results of a survey and case study
Source: Trials. 2025 Jan 21;26:25. doi: 10.1186/s13063-024-08687-5 (PMC11753093; doi:10.1186/s13063-024-08687-5)

Appendix 6 – Screen shots of how data was presented to participants in round 2 of the Delphi


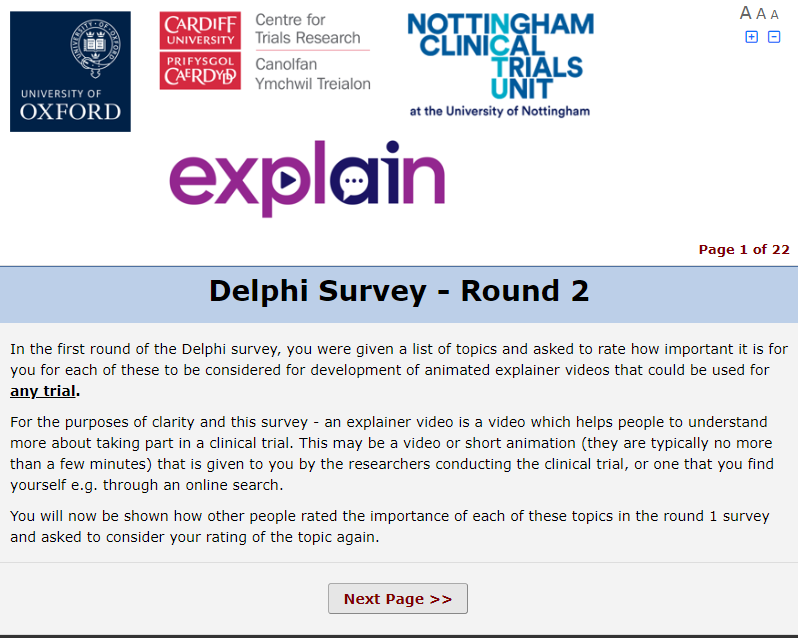


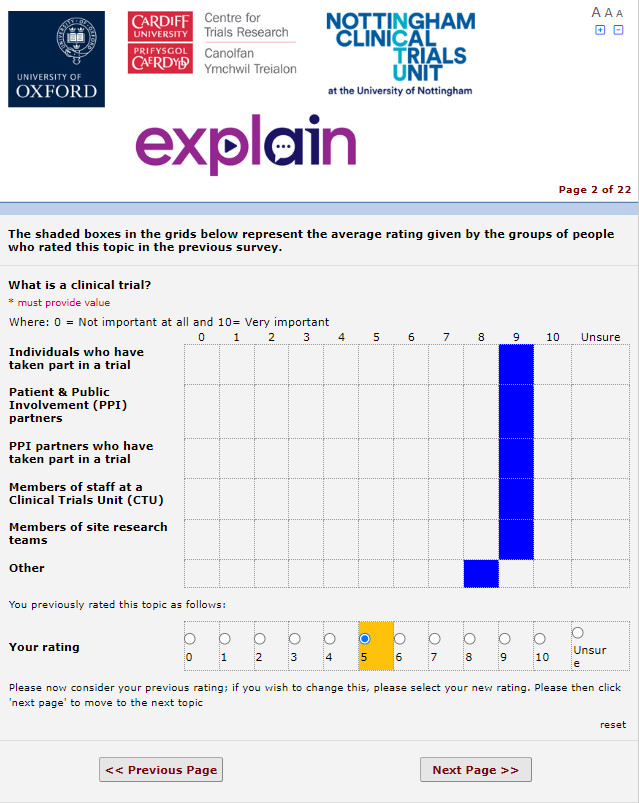


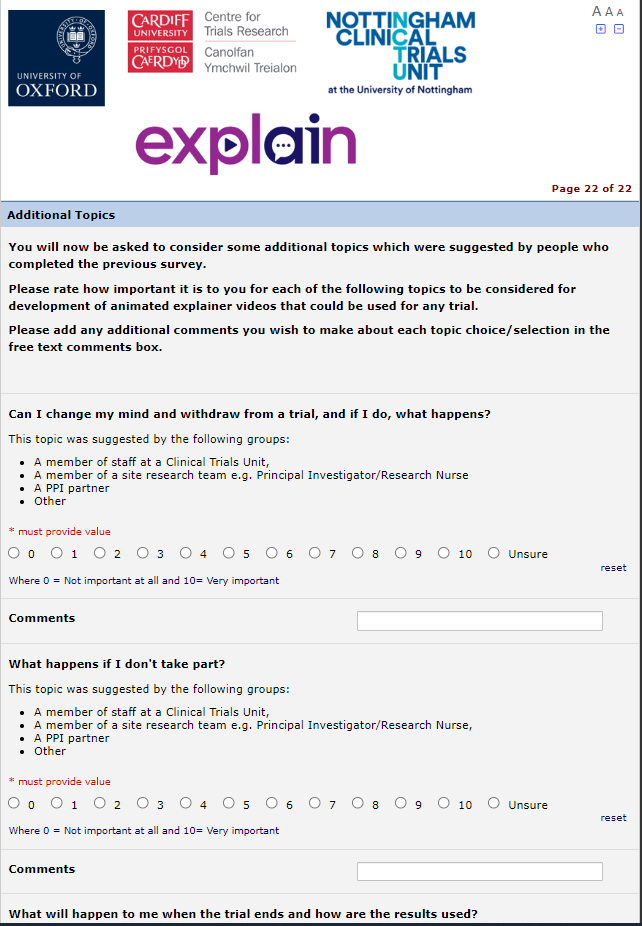

Supplement: Supplementary file 6 — Supplementary Material 6: Appendix 6: Screenshots from round 2. [file 13063_2024_8687_MOESM6_ESM.docx]
