# Supplementary material for: Developing generic clinical trial animated explainer videos in the UK: results of a survey and case study
Source: Trials. 2025 Jan 21;26:25. doi: 10.1186/s13063-024-08687-5 (PMC11753093; doi:10.1186/s13063-024-08687-5)

Appendix 7 – Categorisation of participants for presentation of data in round 2 of the Delphi survey

| **Summary category presented in round 2 of Delphi** | **Responses included in this category** |
| --- | --- |
| Clinical trial participants | “An individual who has participated in a clinical trial” only |
|  | “An individual who has participated in a clinical trial” and “Other” |
| PPI partners | “A PPI partner” only |
| PPI partners who have also taken part in a trial | “An individual who has participated in a clinical trial” and “A PPI partner”. |
|  | “An individual who has participated in a clinical trial” and “An individual who was approached to take part in a clinical trial but decided not to take part” and “A PPI partner”. |
|  | “An individual who has participated in a clinical trial“ and “A PPI partner” and “Other” |
| Members of staff at a CTU | “A member of staff at a Clinical Trials Unit” only |
|  | “A member of staff at a Clinical Trials Unit” and “A member of a site research team e.g. Principal Investigator/Research Nurse”. |
|  | “An individual who has participated in a clinical trial” and “A member of staff at a Clinical Trials Unit” and “Other” |
|  | “An individual who has participated in a clinical trial” and “A member of staff at a Clinical Trials Unit” |
| Members of site research teams | “A member of a site research team e.g. Principal Investigator/Research Nurse” only |
|  | “An individual who has participated in a clinical trial” and “A member of a site research team e.g. Principal Investigator/Research Nurse”. |
|  | “An individual who has participated in a clinical trial” and “An individual who was approached to take part in a clinical trial but decided not to take part” and “A member of a site research team e.g. Principal Investigator/Research Nurse” |
| Other | “Other” only |


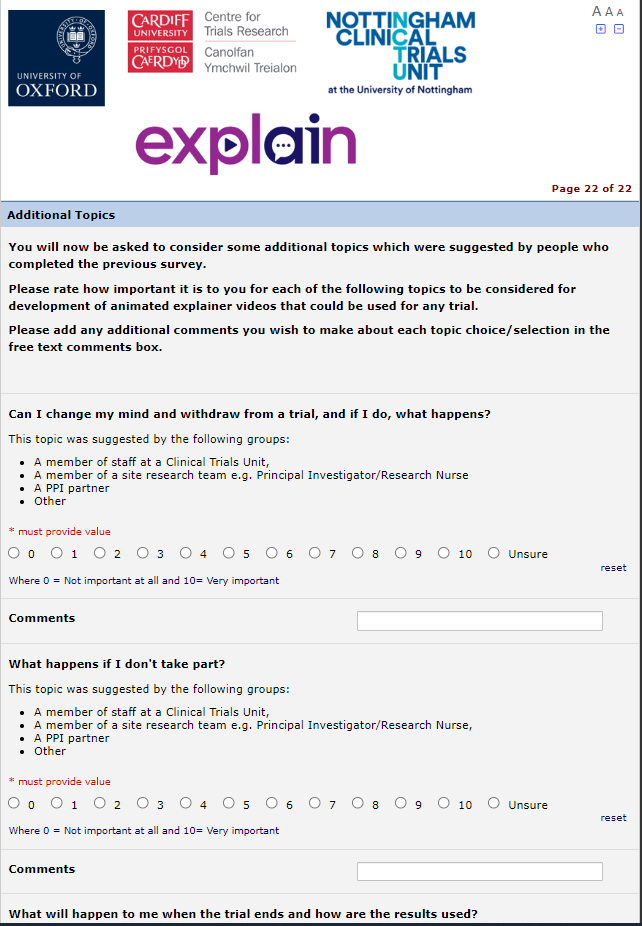

Supplement: Supplementary file 7 — Supplementary Material 7: Appendix 7. [file 13063_2024_8687_MOESM7_ESM.docx]
